# Microplastic Toxicity on Gut Microbiota and Intestinal Cells: Evidence from the Simulator of the Human Intestinal Microbial Ecosystem (SHIME)

**DOI:** 10.3390/toxics13121045

**Published:** 2025-12-02

**Authors:** Xingchao Ren, Chen Su, Yuyan Zhu, James Kar-Hei Fang, Pei Yee Woh

**Affiliations:** 1 Department of Food Science and Nutrition, The Hong Kong Polytechnic University, Hung Hom, Hong Kong SAR, China; xing-chao.ren@connect.polyu.hk (X.R.); chen.su@polyu.edu.hk (C.S.); yuyan.zhu@polyu.edu.hk (Y.Z.); 2 Research Institute for Future Food, The Hong Kong Polytechnic University, Hung Hom, Hong Kong SAR, China; 3 PolyU-BGI Joint Research Centre for Genomics and Synthetic Biology in Global Ocean Resources, The Hong Kong Polytechnic University, Hung Hom, Hong Kong SAR, China; 4 State Key Laboratory of Marine Environmental Health, City University of Hong Kong, Kowloon Tong, Hong Kong SAR, China; 5 College of Health Sciences, VinUniversity, Vinhomes Ocean Park, Gia Lam, Hanoi 100000, Vietnam

**Keywords:** microplastics (MPs), gut microbiota, SHIME, oxidative stress, polystyrene (PS)

## Abstract

Microplastics (MPs) have become widespread environmental contaminants, with increasing evidence of their harmful impacts on human health. MPs generally enter the human body via ingestion, inhalation, or dermal exposure, with the gastrointestinal tract acting as a crucial entrance route. This work utilized the SHIME system to evaluate the effects of polystyrene (PS) MPs on gut microbiota and short-chain fatty acid (SCFA) metabolism in distinct colonic areas. The results demonstrated regional and individual-specific variations in microbial diversity, significant shifts in *Firmicutes*/*Bacteroidetes* (F/B) ratio, and declines in beneficial bacteria, such as *Bifidobacteriaceae*. Moreover, SHIME supernatants were then tested with a co-cultured cell model (Caco-2/HT29-MTX-E12). Results indicated a deteriorative effect on the intestinal model, characterized by enhanced oxidative stress and mitochondrial malfunction. No significant effect on intestinal barrier integrity or mucus secretion was detected. These findings highlight the potential systemic toxicity of PS-MPs on human gut microbiota-mediated mechanisms, emphasizing the necessity for immediate mitigation efforts.

## 1. Introduction

Global waste from plastics presently amounts to 3.5 million metric tons annually. Assuming the trend continues, this figure is anticipated to rise to 25,000 million metric tons by 2040 [1]. Plastic pollution, largely originating from microplastics (MPs) measuring between 1 and 5000 µm [2], has been identified as a global health issue. Numerous studies have focused on the effects of MPs on the growth, development, and immunological responses of aquatic organisms, including horseshoe crabs, mussels, zebrafish, and marine medaka [3,4,5,6].

MPs have been identified in various human biological specimens, including the lungs, liver, brain, placenta, breast milk, blood, sputum, colon, saliva, feces, urine, testis, and semen [7,8,9,10,11,12]. MPs can reach the human body via three primary routes: direct dermal exposure, inhalation, and ingestion from different contaminated food and drinks, including seafood, table salt, and drinking water [13]. MPs have been found to disrupt the healthy balance of gut microbiota, altering intestinal barrier integrity, digestion, metabolism, and immunological function [14,15].

Polystyrene (PS) is a commonly employed polymeric polymer in diverse applications, including food packaging and technology devices. The health consequences of PS-MPs have mostly been studied in experimental animal models due to their widespread usage and environmental diffusion. Nonetheless, the comprehension of the bidirectional connections between MPs consumed by human and their gut microbiota remain restricted. This has emerged as a significant research priority, given that nearly 70% of human bacteria inhabit the colon area.

Colon models have emerged as a crucial and pragmatic target for MP toxicity research, because of the physiological and microbial differences between rodents and humans. The Simulator of the Human Intestinal Microbial Ecosystem (SHIME), established in 1993, is a dynamic, multicompartmental in vitro model that emulates the human gastrointestinal tract [16]. This approach allows the controlled examination of gut microbiota interactions with exogenous chemicals, such as MPs, in settings that closely replicate human intestinal microecology. This work used the SHIME system to investigate the impact of PS-MP exposure on the human gut microbiota and its metabolites in the ascending, transverse, and descending colons. Supernatants from these compartments were collected and evaluated for potential effects by incubating them with cellular models to elucidate the region-specific consequences of exposure to MPs on gut microbial composition and functions.

## 2. Methods

### 2.1. Design and Establishment of the SHIME System

**Figure 1:**
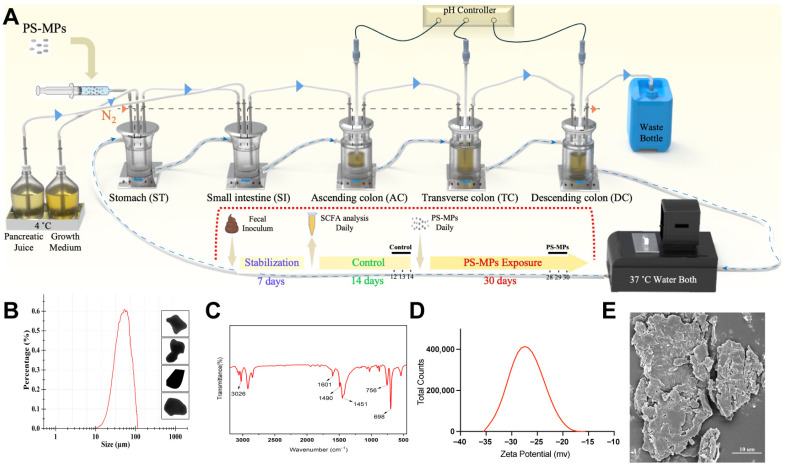
The SHIME system and the characterization of PS-MPs. (**A**) Schematic diagram and experiment design of the SHIME system exposed to PS-MPs. Control group: days 12, 13 and 14 of the control period; PS-MPs group: days 28, 29 and 30 of the PS-MP exposure period. (**B**) Circle equivalent diameter distribution and shape of plastic particles by particles analyzer. (**C**) FTIR spectroscopy of PS-MPs. (**D**) The zeta potentials of PS-MPs. (**E**) SEM image of plastic particles. Scale bar, 10 µm.

The experiment design of the Twin L-SHIME system exposed to PS-MPs was established according to the manufacturer’s manual (Prodigest, Gent, Belgium) (Figure 1A). It consists of two independent SHIME units, each featuring five double-wall biovessels that simulate the stomach (ST), small intestine (SI), ascending colon (AC), transverse colon (TC), and descending colon (DC). The five vessels are connected in sequence through rubber pipes and the suspension in the vessels can be transferred by pumps. Each vessel carried a magnet that provided continuous stirring via a magnetic stirrer. Anaerobic conditions were maintained in the system by flushing it with nitrogen once a day. The temperature was maintained at 37 °C using a circulating water bath. In order to simulate physiological conditions, the pH of the three colon vessels was meticulously adapted using HCl (0.5 M) and NaOH (0.5 M). The pH values that were obtained were as follows: 5.7–5.9 in AC, 6.15–6.4 in TC, and 6.6–6.9 in DC throughout the experiment.

The sterile growth medium (15.6 g powder per liter of deionized water, product code PDP001B, ProDigest, Gent, Belgium) was adjusted to pH 2 using 37% HCl. The quantities of growth medium for the three colon vessels were as follows: 500 mL for AC, 800 mL for TC, and 600 mL for DC. The growth medium was administered to the ST vessel thrice daily at specified intervals and was subsequently transferred to an SI vessel with 60 mL of pancreatic juice containing 12.5 g/L of sodium bicarbonate (Sigma-Aldrich, St. Louis, MO, USA), 6 g/L of Oxgall (Difco, Detroit, MI, USA), and 0.9 g/L of pancreatin (Sigma-Aldrich, St. Louis, MO, USA). Following the accumulation of liquid inside the SI vessel, the entire volume of fluid was progressively pushed through the tube line. This process was repeated until the trash bottle was reached.

### 2.2. Artificial Production and Characterization of PS-MPs

Food-grade PS spoons were purchased from Haojiajie Industrial Limited Company (Shenzhen, China). The spoons were ground into PS-MPs using freezing grinding technology according to the operation manual (CyroMill, Retsch, Haan, Germany). The particles analyzer showed a circle-equivalent diameter distribution of D[v, 0.1]: 23.71 µm, D[v, 0.5]: 55.2 µm and D[v, 0.9]: 113.4 µm, and that PS-MPs were irregular in shape (Figure 1B) (Morphology 4, Malvern Panalytical, Malvern, UK). The distinctive absorption peaks were detected using an FTIR spectrometer (Spotlight 400, PerkinElmer, Waltham, MA, USA) (Figure 1C), and the zeta potentials of PS-MPs measured −27.1 ± 3.13 mV (Figure 1D) (ZEN3600 Zetasizer, Malvern Panalytical, Malvern, UK). The surface microstructure of PS-MPs (Figure 1E) was examined using scanning electron microscopy (SEM) (MAIA3, TESCAN, Brno, Czech Republic). Collectively, these findings confirmed that the manufactured particles were PS plastic fragments at the microscale.

### 2.3. Fecal Inoculum and Initial Composition of Gut Microbiota

Two fresh fecal samples were obtained from healthy adult donors (adult 1 and adult 2) aged 18 and 45 years, respectively, who had resided in Hong Kong for an extended period. Neither donor had a history of antibiotic therapy and drug use within the preceding three months. Upon collection, the samples were immediately placed in an oxygen-free tight container created by anaerobic bags (AnaeroGen 2.5 L, Thermo Scientific, Waltham, MA, USA). A 20 g feces sample was then suspended in 100 mL of anaerobic phosphate buffer (mixture of 8.8 g/L dipotassium phosphate, 6.8 g/L monopotassium phosphate, 0.1 g/L sodium thioglycolate, and 15 g/L sodium dithionite) (Sigma-Aldrich, St. Louis, MO, USA) in a stomacher bag and homogenized for 10 min. After centrifugation, the supernatant was inoculated into the SHIME system within one hour of preparation.

The baseline composition of gut microbiota in fecal inoculum was analyzed. Alpha diversity analysis revealed that the Chao index and Simpson index for adult 1 and adult 2 were 241.14 and 268.20, and 0.07 and 0.10, respectively. Adult 1 and adult 2 had analogous gut bacterial makeup at the phylum level, mainly *Firmicutes*, *Bacteroidetes,* and *Actinobacteria* (Figure S1A), predominated over by the family population of *Ruminococcaceae*, *Bacteroidaceae*, *Lachnospiraceae*, *Veillonellaceae* and *Tannerellaceae* (Figure S1B).

### 2.4. Timeline of PS-MP Exposure and Sample Collection

The SHIME experiment was implemented for 51 days. Upon inoculation of fecal supernatant, we allowed a stabilization period of 7 days for fecal microbes to adapt towards a stable in vitro environment. Following the stabilization period, a 14-day control period ensued, following which we began the 30-day exposure period. We administered a 30 mg PS-MP suspension (dissolved in sterilized distilled water containing 0.01% (*w*/*v*) Tween 80) into ST at 09:00 a.m. everyday for 30 days to replicate human dietary MPs. Supernatant was taken on days 12, 13, and 14 during the control period for the control group, while supernatant was obtained on days 28, 29, and 30 during the exposure periods for the PS-MP group. Daily samples were obtained from 3 vessels throughout the control and exposure periods to assess the operational status of SHIME.

### 2.5. Short-Chain Fatty Acids (SCFAs) Analysis

Gas chromatography (GC, 7980B, Agilent Technologies, Santa Clara, CA, USA) was utilized to analyze the SCFAs (acetic acid, propionic acid, butyric acid, n-butyric acid, valeric acid, and n-valeric acid) that were present in suspensions of three colon vessels. Following a centrifugation of the suspension at 8000 rpm for 10 min, the pH of the supernatant was brought down to 2–3 by adding 1 M HCl. A flame ionization detector (FID, Agilent Technologies, Santa Clara, CA, USA) and DB-FFAP fused-silica capillary column (30 m × 0.32 mm, 123-3232, Agilent Technologies, Santa Clara, CA, USA) were utilized for the GC analysis.

### 2.6. 16S rRNA Sequencing and Analysis

The SHIME solution was used to extract total DNA, and the FastDNA^®^ Spin kit (MP Biomedicals, Santa Ana, CA, USA) and the TruSeq DNA Sample Prep kit (Illumina, San Diego, CA, USA) were utilized for this extraction process. The concentration and purity of the extracts were evaluated with the support of the Thermo Scientific NanoDrop 2000. Polymerase chain reaction (PCR) was utilized in order to amplify the V3–V4 DNA region. The primers 338F and 806R, which are specified in Table S1, were utilized accordingly. After being isolated and purified, the products of the PCR were then measured. After that, the purified amplified fragments were sequenced with the MiSeq PE300 platform (Illumina, San Diego, CA, USA). It was determined that a single operational taxonomic unit (OTU) was formed from sequences that exhibited a similarity of more than 97%.

### 2.7. SEM Sample Preparation and Image Acquisition

The suspensions were immobilized on supports on day 30 of the exposure period by combining 0.1 M PBS buffer with 5% glutaraldehyde for four h. An ethanol series was then used to dehydrate the samples for 10 min each. Two 10 min exposures to absolute ethanol (>99% *v*/*v*) were then performed, and the samples were finally dried for the night. The sample surface was coated with gold particles (Nanoimages, MCM-200, Lafayette, CA, USA) and attached to the holders’ corners with conductive adhesive prior to scanning. Images were taken using a TESCAN MAIA3 scanning electron microscope (magnification = 500× to 15,000×, voltage = 20 kV).

### 2.8. Raman Spectroscopy

A Renishaw micro-Raman spectroscope (Renishaw Plc., Old Town, Wotton-under-Edge, Gloucestershire, UK) was used to acquire the Raman spectra of particles that had been dried on a stainless-steel filter. The spectrum of the silicon wafer was obtained for quality control purposes to calibrate the Raman shift offset. A 785 nm laser with 1200 l/mm grating were chosen for MP characterization, covering a spectral range of 400 to 3600 cm^−1^. Samples were analyzed at 1% laser intensity using a 50× objective with an exposure duration of 10 s. Optical images of particles viewed in a Raman microscope were obtained in bright field illumination. Baseline correction was performed using the automated correlation feature of the Raman system software WiRE 5.3.

### 2.9. Cell Culture

The human colon adenocarcinoma cell line (Caco-2) was acquired from the American Type Culture Collection (ATCC) while the human colorectal adenocarcinoma cell line of mucus-secreting subclone (HT29-MTX-E12) was purchased from Bohui Biotechnology Limited Company (Guangzhou, China). The model of gut epithelium permeability was established in a 9:1 (Caco-2:HT29-MTX-E12) ratio as previously described [17]. Caco-2/HT-29-MTX-E12 cell was conducted using high glucose Dulbecco’s Modified Eagle Medium (DMEM), supplemented with 10% fetal bovine serum, 1% Non-essential amino acids Solution, 1% GlutaMAX and 1% penicillin/streptomycin (Thermo Fisher Scientific, Waltham, MA, USA).

### 2.10. Cell Viability

After being sown in 96-well plates, the cells were cultivated for 24 h. Following inoculation with culture conditions containing various concentrations of AC, TC, and DC supernatant for 24 h, 10 μL of MTT was added to cell. After 4 h of incubation, DMSO was added to the cell culture media, and a microplate reader (SpectraMax M3, San Jose, CA, USA) was employed to access absorbance at 570 nm.

### 2.11. Reactive Oxygen Species (ROS) Generation Analysis

Cells were seeded at a density of 1 × 10^5^ cells per well in a 12-well plate. During the period of 24 h, these cells were subjected to media that was produced from the supernatant. The obtained cells were washed twice with PBS, following treatment with the DCFH-DA probe (Abcam, ab113851) at a concentration of 10 μM. This treatment was carried out at 37 °C for a duration of thirty minutes. Then, the cells were analyzed using a BD FACSymphony A3 Cell Analyzer (BD Biosciences, Franklin Lakes, NJ, USA).

### 2.12. Mitochondria Membrane Potential (MMP) Analysis

Cells were seeded at a density of 1 × 10^5^ cells per well in a 12-well plate. During the period of 24 h, these cells were subjected to media that was produced from the supernatant. The obtained cells were washed twice with PBS, following treatment with a 10 μM solution of JC-1 dye (Abcam, ab113850). This treatment was carried out at 37 °C for a duration of thirty minutes. Then, the cells were analyzed using the BD FACSymphony A3 Cell Analyzer.

### 2.13. Transepithelial Electrical Resistance (TEER) Analysis

Cells were cultured into the apical compartment of a 12 Transwell plate (SPL, Pocheon-si, Gyeonggi-do, Republic of Korea) featuring a polyester membrane with a pore diameter of 0.4 μm. TEER values of co-cultured models must be assessed bi-daily for 21 days before to the following investigation using an epithelial volt-ohm meter (EVOM2, World Precision Instruments, Sarasota, FL, USA). The value was determined as follows: TEER (Ω·cm^2^) = (TEER (Ω) − TEER_Blank_ (Ω)) × A (cm^2^), where A represents the insert membrane area (0.9 cm^2^). The TEER quantifies the convergence and integrity of monolayers. Experiments were performed only on cell monolayers with TEER values over 200 Ω·cm^2^ on day 21. The cells were cultivated for 24 h using medium obtained from the supernatant of both the control group and the PS-MP group, following which the TEER values were evaluated.

### 2.14. Intestinal Permeability

The inserts were positioned in a 12-well culture plate holding 1.5 mL of warmed Hank’s Balanced Salt Solution (HBSS). HBSS comprising 1 mg/mL FITC-Dextran (485/530 nm) and 0.5 mg/mL lucifer yellow was introduced into the apical compartment. The plate was thereafter placed in a shaker incubator set at 37 °C with a revolving speed of 150 rpm. Every 30 min, 0.1 mL was collected from the basolateral compartment of each well until a duration of 2 h was reached. Subsequent to the extraction of samples, the basolateral compartments were replenished with an equal volume of HBSS, and the plates were put back to the shaking incubator until the subsequent measurement. Fluorescence intensity was measured using a microplate reader to determine target marker amount. The apparent permeability coefficient (*P_app_*, cm/s) was calculated as follows: *P_app_* = dQ/dt × 1/(A × C_O_), dQ/dt represents the steady-state flow of the marker across the membrane over a period of 2 h (mg/s), A denotes the insert membrane area (0.9 cm^2^), and C_O_ indicates the starting concentration of the marker in the apical compartment (mg/mL).

### 2.15. ELISA Analysis

The supernatant of the apical compartment from the co-culture was harvested after incubation for 24 h. Human IL-8 levels in the apical suspension were measured using ELISA kits (LMAI Bio, Shanghai, China).

### 2.16. RNA Isolation and Gene Expression Analysis

A MiniBEST Universal RNA Extraction Kit (TaKaRa Bio Inc., Kusatsu, Shiga, Japan; 9767) was utilized in order to extract total RNA from cultivated cells. Additionally, a PrimeScript RT Reagent Kit (TaKaRa Bio Inc., Kusatsu, Shiga, Japan; RR037A) was utilized in order to generate complementary DNA (cDNA). Utilizing a QuantStudio 7 Flex Real-Time PCR System (Applied Biosystems, Thermo Fisher Scientific, Waltham, MA, USA) and the SYBR Premix Ex Taq II Kit (Takara Bio Inc., Kusatsu, Shiga, Japan; RR039A), quantitative real-time polymerase chain reaction (qRT-PCR) was carried out in order to evaluate the expression of genes. Specific amplification was validated using melting curve analysis, and relative expression levels were determined using the 2^−ΔΔCt^ technique, with GAPDH as the internal reference. All primer sequences used are specified in Table S1.

### 2.17. Data Analysis

SPSS 27.0 software (SPSS Inc., Chicago, IL, USA) was utilized for statistical data analysis. When comparing the two groups (control and PS-MPs), a Student’s *t*-test with two tails and no pairing was utilized to determine the differences between them. *p* values that were lower than 0.05 were considered to be statistically significant. *p* values below 0.05 were deemed significant. Results were presented as means ± standard deviation (SD).

## 3. Result

### 3.1. Effect of PS-MP Exposure on the Gut Microbiota Composition

**Figure 2:**
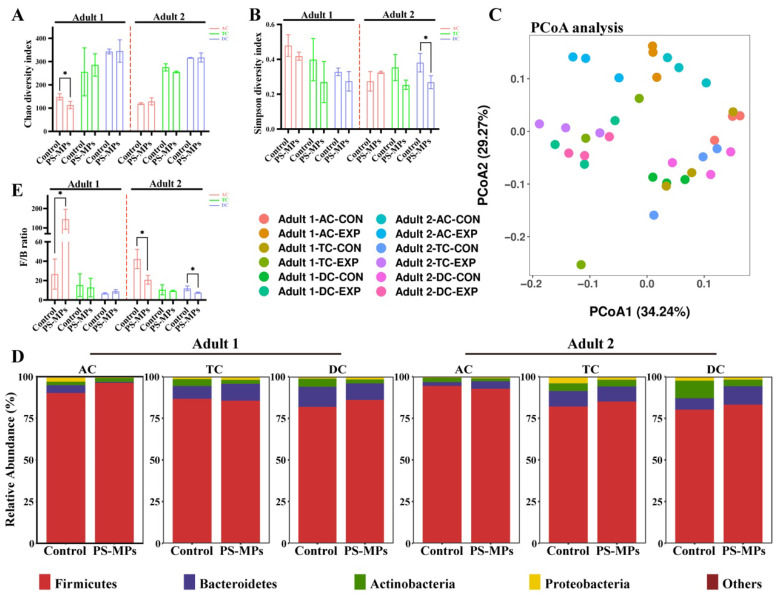
The characterization of SHIME microbiota between the control group and PS-MP group. (**A**,**B**) Alpha diversity is shown by the Chao index (**A**) and Simpson index (**B**). (**C**) Beta diversity determined by principal coordinates analysis. (**D**) The relative abundance of microbiota at the phylum level in the control group and the PS-MP group of AC, TC, and DC in the SHIME system for adult 1 and adult 2. (**E**) The *Firmicutes*/*Bacteroidetes* (F/B) ratio in the control group and PS-MP group of AC, TC, and DC in the SHIME system for adult 1 and adult 2. Data are shown as mean ± SD (n = 3). Statistical significance was calculated by Student’s *t*-test. * *p* < 0.05.

For the microbiota diversity in the SHIME system, the Chao index and Simpson index of alpha diversity were significantly reduced in the PS-MP group of AC in adult 1 and DC in adult 2, respectively (Figure 2A,B). Principal coordinate analysis (PCoA) of beta diversity showed that the microbial communities exposed to PS-MPs were significantly distinguished from those of the control group (Figure 2C). The investigation of microbiota composition revealed that *Firmicutes* and *Bacteroidetes* were the predominant microorganisms (Figure 2D). However, *Firmicutes*/*Bacteroidetes* (F/B) ratio analysis found substantial differences of AC in adult 1, as well as of AC and DC in adult 2 (Figure 2E). Only the F/B ratio of the PS-MP group in the AC of adult 1 was remarkedly increased when compared with the control group. The composition of gut microbiota from both adults at the family level was illustrated in Figure S2, indicating that *Veillonellaceae*, *Bacteroidaceae* and *Lachnospiraceae* were the predominant microorganisms.

LEfSe examination (Figure S3) of the AC, TC, and DC, subsequent to the integration of data from adults 1 and adult 2, revealed significant modifications in the intestinal microbiota composition after treatment with PS-MPs. In all three colon regions, the bacterial genus *Megasphaera* was markedly enriched in the PS-MP group, while beneficial bacteria such as *Lachnoclostridium*, *Bacteroides thetaiotaomicron*, and *Megamonas* were more prevalent in the control group. Notably, phylum *Firmicutes* increased in the DC after PS-MP exposure, meanwhile *Enterobacteriaceae* and *Proteobacteria* were enriched in the control group.

### 3.2. SCFA Products After PS-MP Exposure

**Figure 3:**
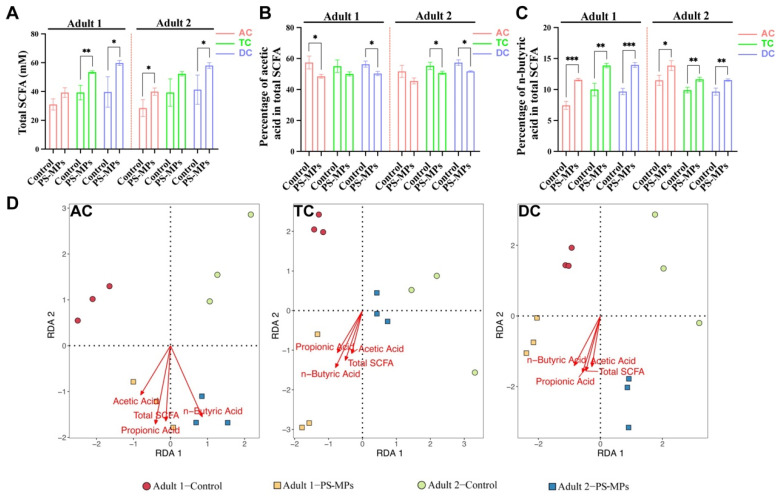
Effect of total SCFA and individual SCFAs between the control group and PS-MP group in the SHIME system. (**A**) The total SCFA in AC, TC, and DC in the SHIME system for adult 1 and adult 2. (**B**,**C**) The percentage of acetic acid (**B**) and n-butyric acid (**C**) within total SCFA in AC, TC, and DC in the SHIME system for adult 1 and adult 2. (**D**) Redundancy analysis (RDA) of microbial composition and SCFAs in the AC, TC, and DC. Data are shown as mean ± SD (n = 3). Statistical significance was calculated by Student’s *t*-test. * *p* < 0.05; ** *p* < 0.01; *** *p* < 0.001.

Figure S4 depicts the overall concentrations of SCFAs and the composition of 6 individual SCFAs inside the SHIME system, assessed during both the stabilization and exposure phases. After PS-MP exposure, the curve of total SCFA concentrations and composition of individual SCFAs in the AC, TC, and DC of 2 adults remained stable with small fluctuations. The total SCFA concentrations were significantly increased after PS-MP exposure in the TC and DC of adult 1, and the AC and DC of adult 2 (Figure 3A). To verify the effect of PS-MP exposure on individual SCFAs, the percentage of individual SCFAs relative to the total SCFA was presented between the control group and PS-MPs group. The proportion of acetic acid in total SCFA generally exhibits a declining trend (Figure 3B). The percentage of propionic acid in the SHIME system is constant except for the DC of adult 2 (Figure S5A). However, the percentage of n-butyric acid strongly increased in three columns of adult 1 and adult 2 following PS-MP exposure (Figure 3C). The percentage of n-valeric acid significantly rose only in the AC and DC of adult 1, and DC of adult 2 (Figure S5B). Meanwhile, PS-MP exposure only affected AC of adult 1 in SHIME system, resulting in an augmentation of the percentage of i-butyric acid and i-valeric acid in total SCFA (Figure S5C,D). Hence, PS-MP exposure could exert a specific influence on the concentrations and compositions of individual SCFAs. RDA analysis indicates that the PS-MP group is markedly distinct from the control group at three sites (Figure 3D). Compared with the control group, acetic acid, propionic acid, butyric acid and total SCFA, shown by arrows, are in proximity to the PS-MP group samples, indicating that gut microbiota exposed to PS-MPs is significantly linked to SCFA production.

### 3.3. Interaction Between PS-MPs and Gut Microbiota in the SHIME System

**Figure 4:**
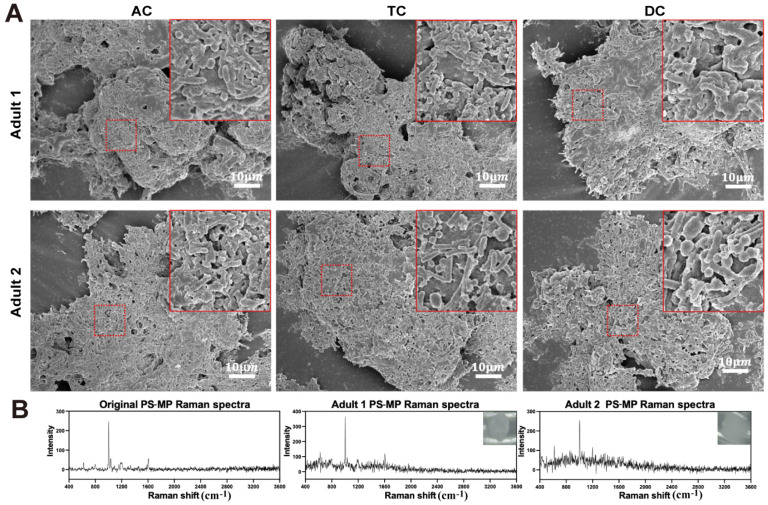
SEM observation and Raman analysis of the interaction between PS-MPs and gut microbiota on day 30 of the exposure period. (**A**) Representative SEM images of the interaction between PS-MPs and gut microbiota in AC, TC and DC within the adult 1 and adult 2 SHIME systems on day 30 of the exposure period. (**B**) Raman spectra of original PS-MPs and PS-MPs exposed to DC of the SHIME system at day 30.

To evaluate the bidirectional effects between PS-MPs and gut microbiota, we performed SEM and Raman spectroscopy on day 30 of PS-MP exposure. We observed a considerable number of gut microorganisms, with bacillus and cocci predominantly colonizing the surface of PS-MPs in AC, TC, and DC (Figure 4A). However, there was no alteration in the physicochemical parameters of the PS-MPs surface following 30 days of exposure (Figure 4B).

### 3.4. Effects of SHIME Supernatants on Caco-2/HT29-MTX-E12 Co-Culture Cells

To confirm the potential toxicity of the SHIME supernatant from the control group in Caco-2/HT29-MTX-E12 cells, we conducted an MTT assay using supernatant diluted in ratios of 2:1, 5:1, 10:1, 20:1 and 40:1. The results showed the absence of toxicity at dilutions of 20× and 40× (Figure S6). Subsequent experiments employed 20× supernatant to treat cells.

**Figure 5:**
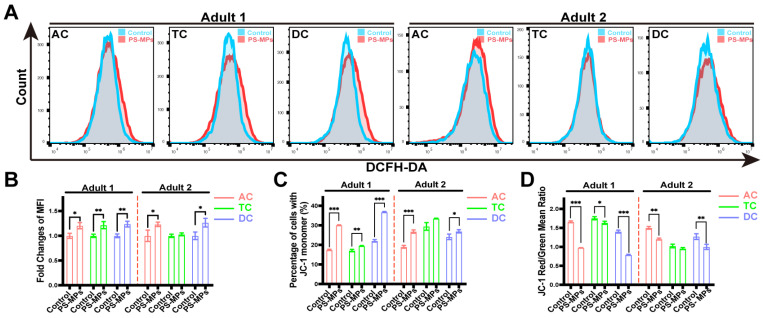
Analysis of ROS levels and mitochondrial function in the Caco-2/HT29-MTX-E12 co-culture model using the SHIME supernatant. (**A**,**B**) Flow cytometry analysis of ROS levels (**A**) and mean fluorescence intensity (MFI) (**B**) in a co-culture cell model treated with the supernatant from the control and PS-MP groups. (**C**) The quantified percentage of cells with JC-1 monomer between the control and PS-MP groups. (**D**) Red-to-green mean ratio of JC-1 staining. Data are shown as mean ± SD (n = 3). Statistical significance was calculated by Student’s *t*-test. * *p* < 0.05; ** *p* < 0.01; *** *p* < 0.001.

DCFH-DA probe and JC-1 staining were utilized as indicators for ROS levels and MMP to further evaluate the effects of the SHIME supernatant. The formation of ROS and the mean fluorescence intensity (MFI) of cells stimulated with the PS-MP supernatant was significantly increased in the SHIME system, except for TC in adult 2 (Figure 5A,B). The MMP of cells was remarkably diminished after incubating with the PS-MP supernatant, with the exception of TC in adult 2, showing that it caused mitochondria damage (Figure 5C,D).

**Figure 6:**
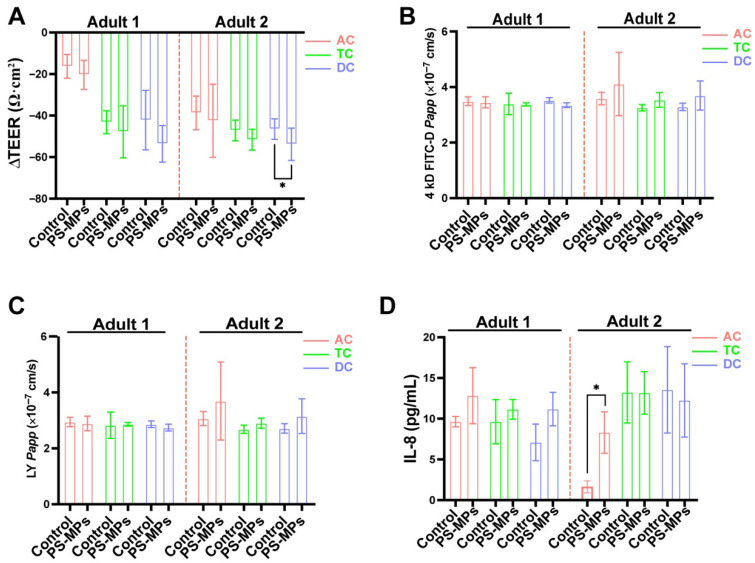
Effect of the SHIME supernatant on intestinal barrier integrity in Caco-2/HT29-MTX-E12 co-culture model. (**A**) ΔTEER values measurement of co-culture cell model treated with the supernatant from the control and PS-MP groups. (**B**,**C**) *Papp* calculation of 4 kD FICT-D (**B**) and LY (**C**) in the co-culture cell model treated with the supernatant from the control and PS-MPs groups. (**D**) IL-8 concentration of supernatant in apical compartment of co-culture cells. Data are shown as mean ± SD (n = 3). Statistical significance was calculated by Student’s *t*-test. * *p* < 0.05.

The assessment of TEER and the flux of fluorescent molecules through the cells’ monolayers were employed to confirm intestinal barrier integrity. ΔTEER serves as an indicator of the variation in TEER values before and after PS-MP exposure. ΔTEER values exhibited no significant alterations in SHIME supernatant, with the exception of a significant decrease in DC of adult 2 following PS-MP exposure. (Figure 6A). To further test the intestinal permeability, the diffusion of 4kD FITC-D and LY in the epithelial cell monolayers was measured. Permeability showed no notable variations between the control and PS-MP groups across all areas (AC, TC, and DC) in adult 1 and adult 2 (Figure 6B,C). At the same time, there were no statistically significant differences in either donor with regard to the expression levels of tight junction-associated genes. These genes include ZO-1, OCLN, and CLDN1. Furthermore, the levels of expression of mucus secretion-related genes, MUC5AC and MUC2, were constant, and there were no significant differences seen in any colonic region between the two groups (Figure S7).

The supernatant from the apical compartment of the Transwell was collected to measure the IL-8 level. IL-8 levels were significantly increased in the AC of adult 2 after PS-MP exposure, whereas other regions exhibited no notable changes (Figure 6D). Nonetheless, an upward trend of IL-8 level was observed under the influence of supernatant from the AC, TC and DC of adult 1.

## 4. Discussion

As MPs are considered non-toxic, they were, for a long time, deemed irrelevant to human health. However, a potential detrimental effect of MPs on gut health has been proposed [11]. At present, there is a scarcity of research regarding the effects and toxicity of MPs in intestinal models. This in vitro experiment utilizing the SHIME system combined with a cell model to systematically evaluate the impact of MPs exposure on intestinal microbiota structure, SCFA metabolism, and its effects on cellular oxidative stress and mitochondrial function, revealing the complex toxic effects induced by MPs in different intestinal regions. Recent exposure assessments estimate that humans may ingest approximately 0.1–5 g of microplastics per week, equivalent to 14–714 mg per day, depending on diet, drinking water source, and environmental contamination levels [18]. Based on this range, the 30 mg/day MPs dose applied in the present study represents an environmentally relevant lower-bound exposure scenario.

Numerous studies using animal models has suggested that ingesting MPs may cause an imbalance in the gut microbiota [19]. The F/B ratio serves as a crucial measure of the structural integrity of bacterial communities. Obesity corresponds to a high F/B ratio, while inflammatory bowel disease (IBD) is linked to a low F/B ratio. Research indicates that the F/B ratio in mice elevated after polyethylene (PE), polyethylene terephthalate (PET), and polypropylene (PP) MPs treatment, whereas it decreased in groups exposed to PS and polyvinyl chloride (PVC) MPs [20]. After exposure to MPs, the F/B ratio significantly rose in the AC of adult 1 and reduced in the AC and DC of adult 2, indicating notable regional disparities in this investigation. Although the baseline F/B ratios of adults 1 and 2 were almost identical at the phylum level (Figure S1A), significant differences remained at the family level (Figure S1B). *Firmicutes* of adult 1 were primarily dominated by *Ruminococcaceae* and *Lachnospiraceae*, while microbiota of adult 2 featured a more prominent proportion of the *Bacteroidaceae*, *Prevotellaceae* and *Rikenellaceae* families. These subtle compositional differences may have influenced how each microbial community adapted to PS-MPs exposure, contributing to the opposite F/B responses observed in the two individuals.

Currently, several in vitro intestinal simulation models have been used to investigate the effects of various plastic polymers on the microbiota that are found in the digestive tract. To examine the effects of MPs, the mucosal artificial colon model has been used [21,22]. The study reported that exposure to MPs in this model led to an increased prevalence of human pathogenic bacteria *Enterobacteriaceae* and *Desulfovibrionaceae* [22], which, in contrast with our findings, observed a reduction in their abundance. This discrepancy may be attributable to elevated concentrations of butyric acid, which inhibited the proliferation of pro-inflammatory bacteria in our model [23]. In the Simgi^®^ system simulating gut, exposure to MPs resulted in distinct alterations in gut microbiota composition among different individuals. Specifically, Tamargo et al. observed an increased abundance of *Firmicutes* and *Desulfobacterota* in adult 1, whereas adult 2 exhibited a decreased number of *Bacteroidetes* and *Proteobacteria* in the AC. Additionally, a notable reduction in viable *Bifidobacterium* was observed across the AC, TC, and DC compartments [24]. Our intestinal simulation model demonstrated elevated relative abundances of the family *Ruminococcaceae* and *Lachnospiraceae* during MPs exposure. Meanwhile, there was a notable reduction of *Proteobacteria* in the AC of adult 1, as well as in the TC and DC of adult 2, and the abundance of *Bifidobacteriaceae* was significantly diminished in the AC, TC, and DC of adult 2. The study similarly revealed that MPs induced a notable reduction in the abundance of *Bifidobacteriaceae* in mice [25]. Additionally, MPs aggravated microbial community disruption in the radiation-affected small intestine of mice, evidenced by the reduction of microbiota such as *Lactobacillaceae*, *Muribaculaceae*, and *Bifidobacteriaceae* [26].

Furthermore, exposure to MPs in human gut microbiota resulted in an impressive rise in *Megasphaera* abundance in our study, consistent with findings on polylactide (PLA) MPs [27]. Presently, limited research has been conducted on *Megasphaera*, which may function as a possible biomarker for lung cancer [28]. Different *Megasphaera* species possess specific lactate utilization strategies, producing butyrate via the reverse β-oxidation (rBOX) pathway or propionic acid via the acrylic acid pathway, thus playing a key pivotal role in the SCFA metabolic network [29]. Furthermore, *M. indica* was shown to cross-feeding rely on lactic acid produced by *Bifidobacterium*, significantly promoting butyrate production, suggesting that its enrichment reflects the remodeling of the microbial community’s functional structure [30]. More concerningly, some *Megasphaera* species (such as *M. elsdenii*) can disrupt intestinal epithelial homeostasis and increase permeability in animal models and activate DC-mediated Th1/Th17 immune responses through TLR4/NF-κB, thereby exacerbating colonic inflammation and tumorigenesis [31]. Based on the above evidence, the enrichment of *Megasphaera* after MPs exposure may not only lead to changes in SCFA composition, but may also reflect altered substrate availability, remodeling of the microbial cross-feeding network, and potential activation of mucosal inflammatory signals. Therefore, *Megasphaera* enrichment may be a key node in MPs-induced intestinal metabolic stress and immune perturbation, warranting further investigation into its causal relationship in future studies.

The alterations in SCFA composition observed in this study, with elevated butyrate accompanied by reduced acetate, may indicate metabolic reprogramming of the gut microbiota in response to MPs exposure. On the one hand, butyrate, as an important metabolite for maintaining epithelial energy supply and barrier homeostasis, may represent a compensatory adjustment by the microbiota under stress to support mucosal function [32]. On the other hand, acetate is an important substrate for cross-feeding among various microbial communities, and its decrease suggests a disturbance in the microbial metabolic network, possibly reflecting reduced carbon flow allocation efficiency or increased ecological pressure [33]. Therefore, this metabolic pattern of butyrate and acetate differentiation may carry certain protective significance while also indicating a state of community stress, reflecting the complex metabolic response of the microbiota under MPs exposure.

Our SEM results clearly revealed the colonization of large numbers of microorganisms on MP surfaces. MPs possess a durable solid surface that offers a distinctive colonization framework for microbes, creating a unique biological niche that facilitates biofilm development, referred to as the “plastisphere” [34]. MP biofilm provides physical support, creating a stable habitat for microorganisms, protecting them from external environmental factors, and promoting the proliferation and dissemination of bacteria [34]. The study indicated that biofilms on MPs in different environments can enrich pathogenic microorganisms and are resistant to antibiotics and genes [35]. However, similar in vitro studies of human intestinal models, as well as our study, did not detect significant alterations in the physicochemical properties of MPs using Raman spectroscopy [21,22].

The toxicological effects of MPs exposure emphasize alterations in oxidative stress, barrier properties, cytotoxicity, and immunological response [36]. Exposure to oxidized low-density (Ox-LD) PE-MPs modified the intestinal microbiota composition in mice and induced more pronounced intestinal histological alterations, oxidative stress, and inflammatory responses [37]. The findings indicate that cells subjected to MPs in vitro resulted in oxidative damage and apoptosis [38,39]. A previous study found that MPs induce apoptosis in granulosa cells and impair oocyte quality in ovarian follicles [40]. Both this reproductive toxicity study and our study utilized in vitro systems to reveal the cellular effects of MPs, but the subjects were the reproductive system and the gastrointestinal microbiome, respectively, demonstrating the specific role of MPs in different organs. The key processes that are responsible for the cytotoxicity that is generated by MPs are the buildup of intracellular ROS and the mitochondria depolarization [41]. Presently, there is insufficient evidence about the influence of microbial metabolites on human cell models after the exposure of intestinal microbiota to MPs, with several studies focusing on the effects of MPs on microbiota and related organs in animal models. Additionally, the co-culture cell provided additional confirmation of the metabolic toxicity of the gut as well as of the impact of metabolites on the activity of cells. The exposure of MPs to the SHIME supernatant resulted in a considerable increase in ROS levels and produced mitochondrial membrane depolarization. These findings suggest that oxidative stress and mitochondrial function disruption are primary factors of cell damage.

Direct exposure of MPs to Caco2 cells increased epithelial permeability and decreased the expression of tight junction-related genes [42]. Our research determined that SHIME supernatant does not influence permeability or mucus secretion in co-culture cells, suggesting a negligible effect on the intestinal barrier. The supernatant in a mucosal artificial colon model similarly did not significantly alter the permeability and mucin production of human intestinal cells during exposure of adult and new toddler gut microbiota to MPs [21,22]. It is worth noting that the use of a 20× dilution, while intended to prevent nonspecific cytotoxicity, may somewhat limit the model’s ability to detect very mild or sub-lethal barrier disturbances within the 24 h exposure period. However, a trend of increased mucin gene expression was noted exclusively in some new toddlers [22]. Nonetheless, elevated IL-8 levels were observed in the AC region of adult 2, which was closely related to a decreased F/B ratio and a significant reduction in *Bifidobacteriaceae* after exposure to this region. The decreased F/B ratio, together with the loss of probiotics, weakens mucosal barrier function and immune regulation, rendering the epithelium more susceptible to inflammatory stimulation. IL-8 is essential in intestinal epithelial cells, playing a significant role in inflammatory responses, promoting intestinal barrier repair, and modulating immunological processes. The findings suggest that the influence of AC may require increased scrutiny.

Furthermore, the potential dissolution of chemicals from MPs under simulated intestinal conditions must be considered. Even food-grade PS may release trace amounts of residual monomers, oligomers, or chemical additives, which have been shown to induce oxidative stress and mitochondrial damage even without particulate action [43]. Therefore, the oxidative reactions observed in this study may be influenced by both the particulates themselves and their dissolutions. The widespread presence of MPs and their potential adverse impacts on human health is emerging as a significant public health concern. MPs directly influence the intestinal microbiota and immune system, while their surface chemical characteristics facilitate the efficient adsorption of hazardous environmental substances, including persistent organic pollutants and heavy metals [44,45]. This adsorption enhances the toxicity of MPs to human health and designates them as carriers for the transport and accumulation of environmental contaminants [46]. Thus, in vitro research techniques serve as a crucial tool for clarifying the mechanisms by which MPs and their effects adsorb hazardous chemicals. They can not only precisely simulate human exposure scenarios but also efficiently evaluate their possible health risks. This research is crucial for the formation of public health policies and the development of pollutant risk management techniques. However, this study still cannot reproduce key cross-organ processes such as in vivo immune regulation, liver metabolism, and systemic signaling. Therefore, these results mainly reflect local mechanistic effects, and future studies need to incorporate comprehensive models that include host immunity or metabolic processes to more accurately assess their actual impact on the human body.

## 5. Conclusions

This research systematically evaluated the impact of MPs on gut microbiota composition, SCFA metabolism, and cellular responses using an advanced SHIME in vitro system. MPs exposure induced region- and individual-specific alterations in gut microbial diversity. Exposure to MPs significantly changed the structure of the intestinal microbiota, manifested as regional changes in the F/B ratio. The F/B ratio elevated in the AC of adult 1, whereas the F/B ratio in the AC and DC of adult 2 decreased, indicating variations in the metabolic responses of microbiota between individuals. Metabolic alterations in SCFAs were evidenced by an increase in total SCFAs and the proportion of butyric acid within total SCFAs, alongside a reduction in the percentage of acetic acid in total SCFAs. SEM results confirmed the direct contact between MPs and bacteria, although no substantial changes in the physicochemical properties of MPs were found. Experiments on Caco-2/HT29-MTX-E12 cells confirmed that the SHIME supernatant exposed to MPs caused functional damage at the cellular level by inducing oxidative stress and mitochondrial dysfunction. SHIME supernatant of MPs exposure did not affect intestinal permeability, inflammation, and mucus secretion. Future research should focus further on the cumulative effects of long-term exposure to MPs on the function of the microbiota metabolism and explore the potential systemic toxicity of MPs via gut microbiota-mediated mechanisms to reduce harmful impacts of MPs exposure to host health.

## Data Availability

The raw data supporting the conclusions of this article will be made available by the authors on request. The data used to support the findings of this study have been deposited in the NCBI Sequence Read Archive under Accession No. PRJNA1243730.

## Supplementary Materials

The following supporting information can be downloaded at https://www.mdpi.com/article/10.3390/toxics13121045/s1, Figure S1. The baseline microbiota composition of fecal inoculum from adult 1 and adult 2. (A): The relative abundance of gut microbiota from adult 1 and adult 2 at the phylum level. (B): The relative abundance of gut microbiota from adult 1 and adult 2 at the family level. Figure S2. The relative abundance of gut microbiota at the family level. (A–C): The relative abundance of gut microbiota at the family level in the control group and PS-MPs group of AC (A), TC (B) and DC (C) in SHIME system for adult 1. (D–F): The relative abundance of gut microbiota at the family level in the control group and PS-MPs group of AC (D), TC (E) and DC (F) in SHIME system for adult 2. Figure S3. The differences in gut microbiota composition of different colon regions following PS-MPs exposure. (A–C): LDA score and cladogram of LEfSe analysis in the control group and PS-MPs group of AC (A), TC (B), and DC (C) in both adults. The LDA score threshold was set at 2.0, and statistical differences in taxa abundance were evaluated using the Mann–Whitney test with a significance level of 0.05. Figure S4. Total SCFAs and composition of major SCFAs in the SHIME system. (A–B): The total SCFAs of AC, TC and DC in SHIME system of adult 1 (A) and adult 2 (B) during control and exposure period. (C–E): The composition of major SCFAs of AC (C), TC (D) and DC (E) in SHIME system of adult 1 during control and exposure period. (F–H): The composition of major SCFAs of AC (F), TC (G) and DC (H) in SHIME system of adult 2 during control and exposure period. Figure S5. The percentage of propionic acid (A), n-Valeric acid (B), i-butyric acid (C) and i-valeric acid (D) within total SCFA. Data are shown as mean ± SD (n = 3). Statistical significance was calculated by Student’s *t*-test. * *p* < 0.05; ** *p* < 0.01; *** *p* < 0.001. Figure S6. Effect of SHIME supernatant on cell viability of Caco-2/HT29-MTX-E12 co-culture cell model in the control group. (A–C): Effect of medium containing different dilutions (dilute in ratios of 2:1, 5:1, 10:1, 20:1 and 40:1) of SHIME supernatant from adult 1 AC (A), TC (B) and DC (C) on cell viability in the co-culture cell model. (D–F): Effect of medium containing different dilutions (dilute in ratios of 2:1, 5:1, 10:1, 20:1 and 40:1) of SHIME supernatant from adult 2 AC (D), TC (E) and DC (F) on cell viability in the co-culture cell model. Data are show as mean ± SD (n = 6). Statistical significance was calculated by Student’s *t*-test (two-tailed). * *p* < 0.05; ** *p* < 0.01; *** *p* < 0.001. Figure S7. qRT-PCR quantification of intestinal function genes in Caco-2/HT29-MTX-E12 co-culture cell. (A): Relative mRNA expression of tight-junction related ZO-1, OCLN and CLDN1 genes. (B): Relative mRNA expression of mucus secretion related MUC5AC and MUC2 genes. Data are shown as mean ± SD (n = 3). Statistical significance was calculated by Student’s *t*-test. * *p* < 0.05; ** *p* < 0.01; *** *p* < 0.001. Table S1. The primers used in this study.
