# Supplementary material for: Microplastic Toxicity on Gut Microbiota and Intestinal Cells: Evidence from the Simulator of the Human Intestinal Microbial Ecosystem (SHIME)
Source: Toxics. 2025 Dec 2;13(12):1045. doi: 10.3390/toxics13121045 (PMC12737304; doi:10.3390/toxics13121045)
Supplement: Supplementary file 1 [file toxics-13-01045-s001.zip › toxics-3977491-supplementary.pdf]

Support Information

Microplastic Toxicity on Gut Microbiota and Intestinal Cells: Evidence from the Simulator of the Human Intestinal Microbial Ecosystem (SHIME)

Xingchao Ren [1], Chen Su [1], Yuyan Zhu [1,2], James Kar-Hei Fang [1,2,3,4,*] and Pei Yee Woh [5,*]

1 Department of Food Science and Nutrition, The Hong Kong Polytechnic University, Hung Hom, Hong Kong SAR, China; xing-chao.ren@connect.polyu.hk (X.R.); chen.su@polyu.edu.hk (C.S.); yuyan.zhu@polyu.edu.hk (Y.Z.)

2 Research Institute for Future Food, The Hong Kong Polytechnic University, Hung Hom, Hong Kong SAR, China

3 PolyU-BGI Joint Research Centre for Genomics and Synthetic Biology in Global Ocean Resources, The Hong Kong Polytechnic University, Hung Hom, Hong Kong SAR, China

4 State Key Laboratory of Marine Environmental Health, City University of Hong Kong, Kowloon Tong, Hong Kong SAR, China

5 College of Health Sciences, VinUniversity, Vinhomes Ocean Park, Gia Lam, Hanoi 100000, Vietnam

* Correspondence: james.fang@polyu.edu.hk (J.K.-H.F.); peiyee.w@vinuni.edu.vn (P.Y.W.)

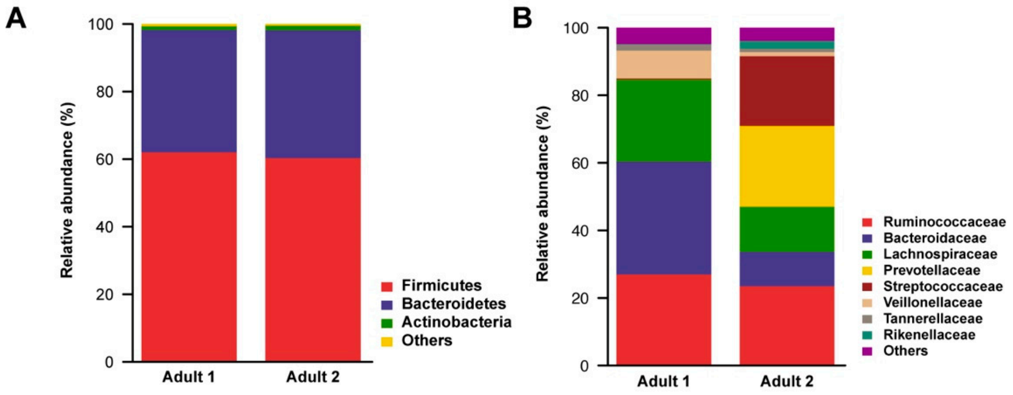

Fig. S1. The baseline microbiota composition of fecal inoculum from adult 1 and adult 2. **A**: The relative abundance of gut microbiota from adult 1 and adult 2 at the phylum level. **B**: The relative abundance of gut microbiota from adult 1 and adult 2 at the family level.

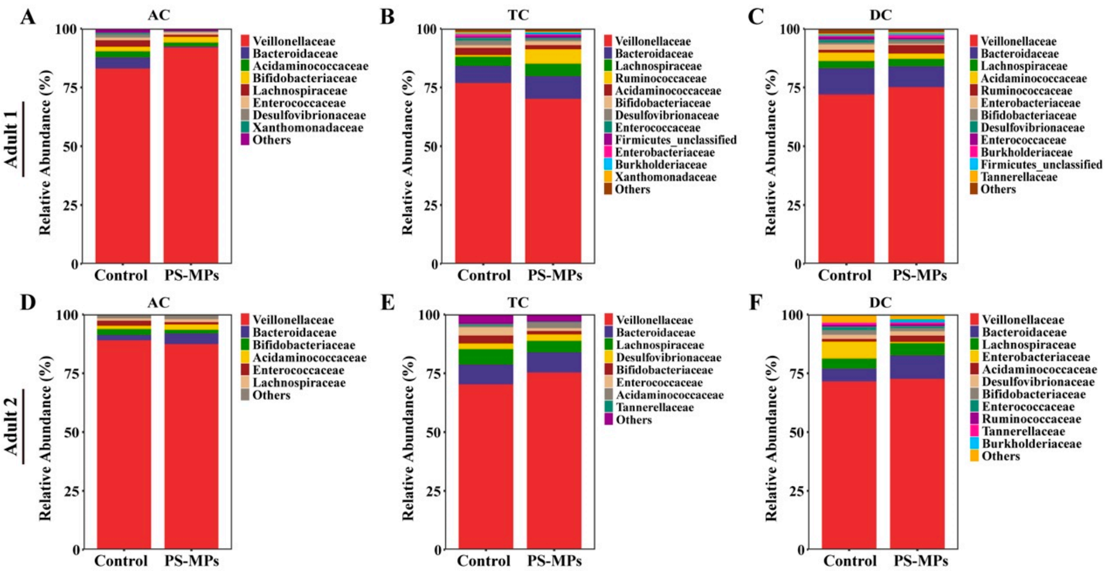

Fig. S2. The relative abundance of gut microbiota at the family level. **A-C**: The relative abundance of gut microbiota at the family level in the control group and PS-MPs group of AC (A), TC (B) and DC (C) in SHIME system for adult 1. **D-F**: The relative abundance of gut microbiota at the family level in the control group and PS-MPs group of AC (D), TC (E) and DC (F) in SHIME system for adult 2.

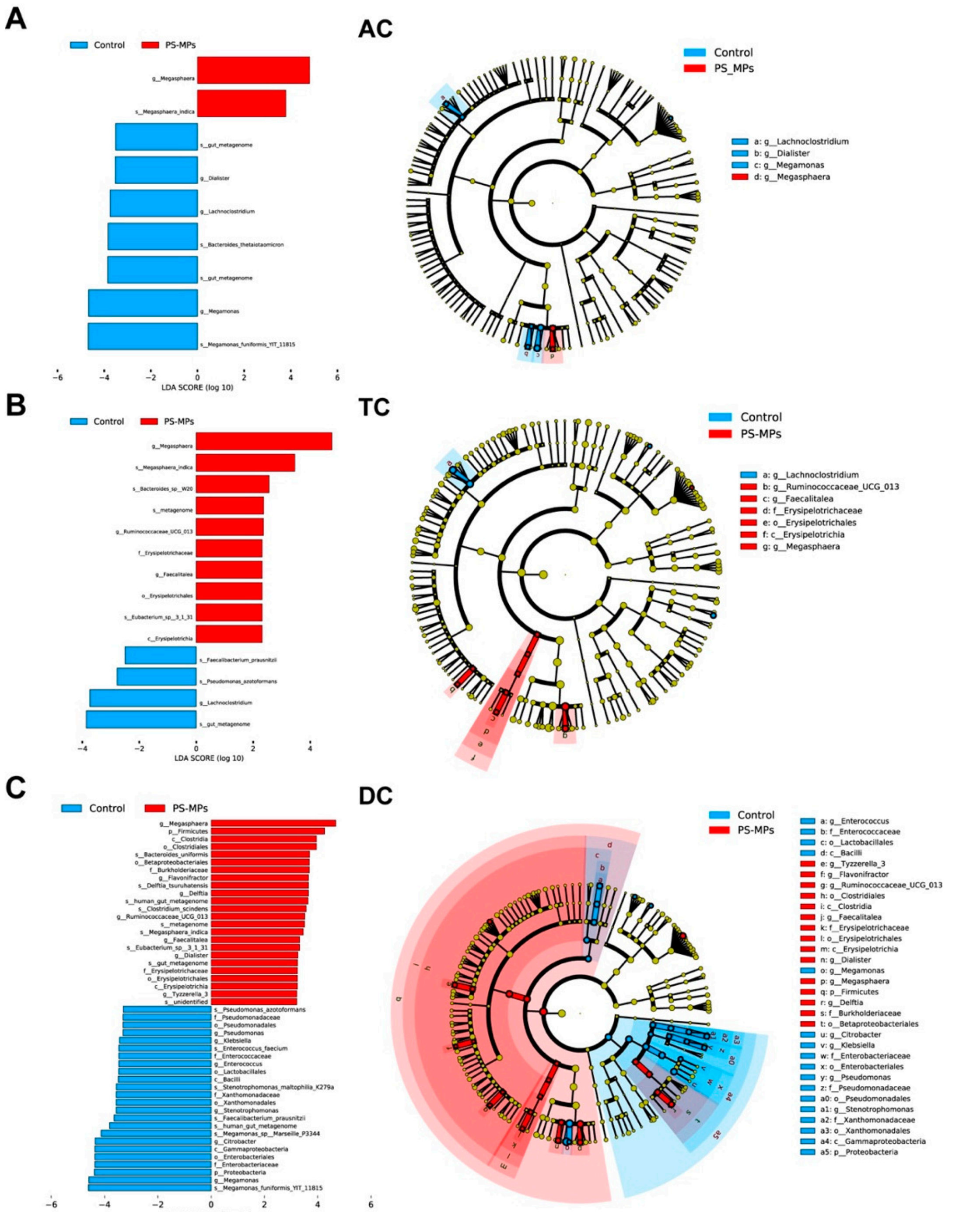

Fig. S3. The differences in gut microbiota composition of different colon regions following PS-MPs exposure. A-C: LDA score and cladogram of LEfSe analysis in the control group and PS-MPs group of AC (A), TC (B), and DC (C) in both adults. The LDA score threshold was set at 2.0, and statistical differences in taxa abundance were evaluated using the Mann–Whitney test with a significance level of 0.05.

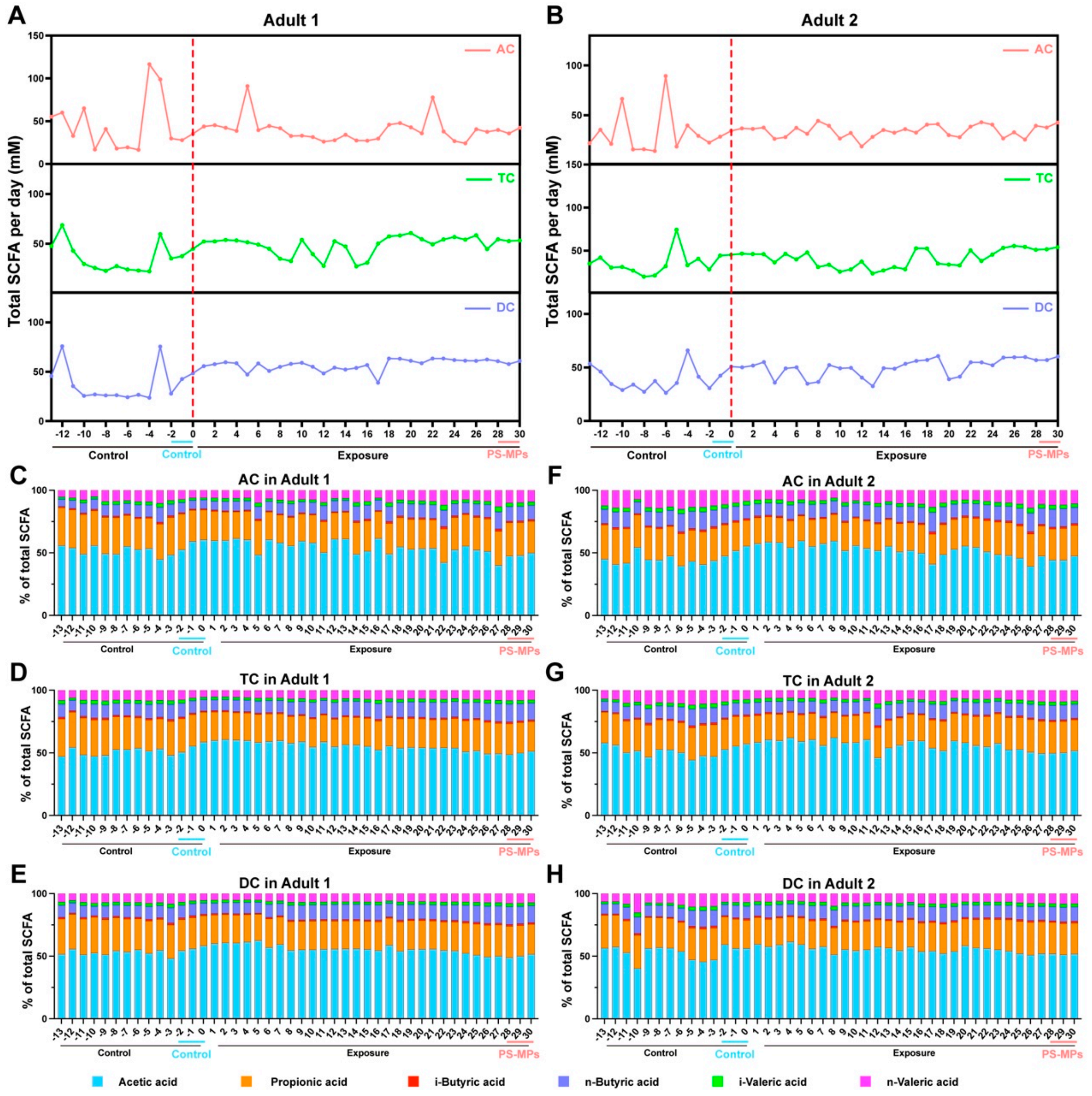

Fig. S4. Total SCFAs and composition of major SCFAs in the SHIME system. **A-B**: The total SCFAs of AC, TC and DC in SHIME system of adult 1 (A) and adult 2 (B) during control and exposure period. **C-E**: The composition of major SCFAs of AC (C), TC (D) and DC (E) in SHIME system of adult 1 during control and exposure period. **F-H**: The composition of major SCFAs of AC (F), TC (G) and DC (H) in SHIME system of adult 2 during control and exposure period.

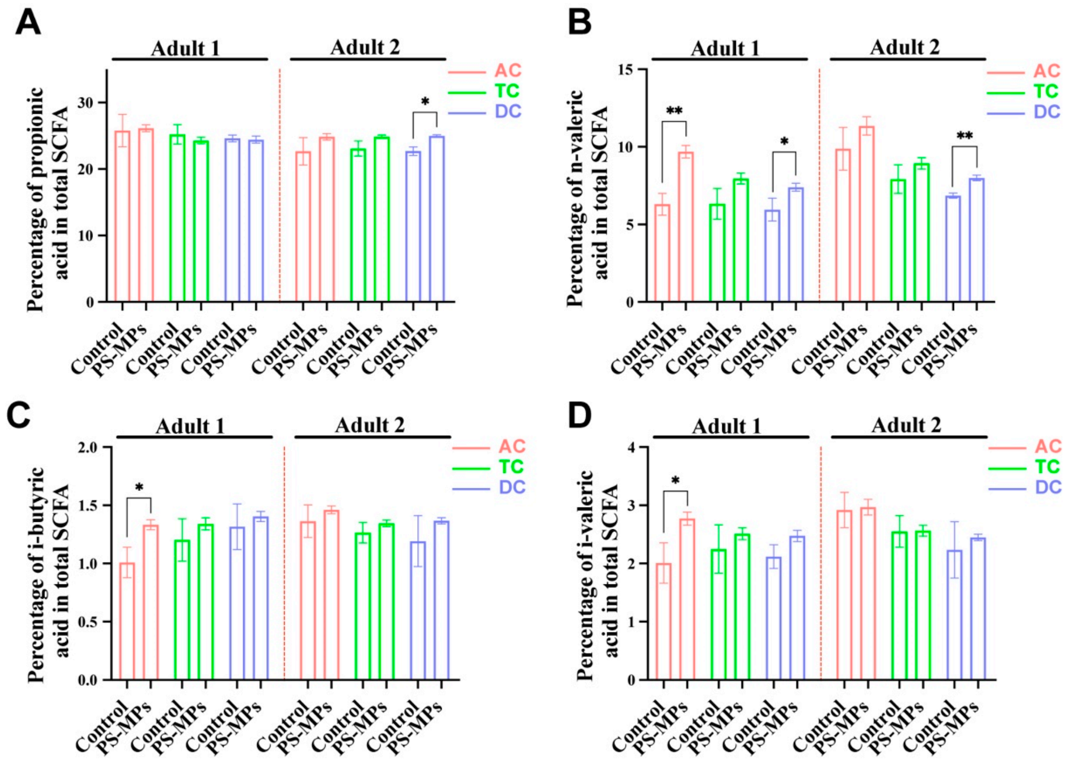

Fig. S5. The percentage of propionic acid (**A**), n-Valeric acid (**B**), i-butyric acid (**C**) and i-valeric acid (**D**) within total SCFA. Data are shown as mean ± SD (n=3). Statistical significance was calculated by Student's t-test. * $p < 0.05$; ** $p < 0.01$; *** $p < 0.001$.

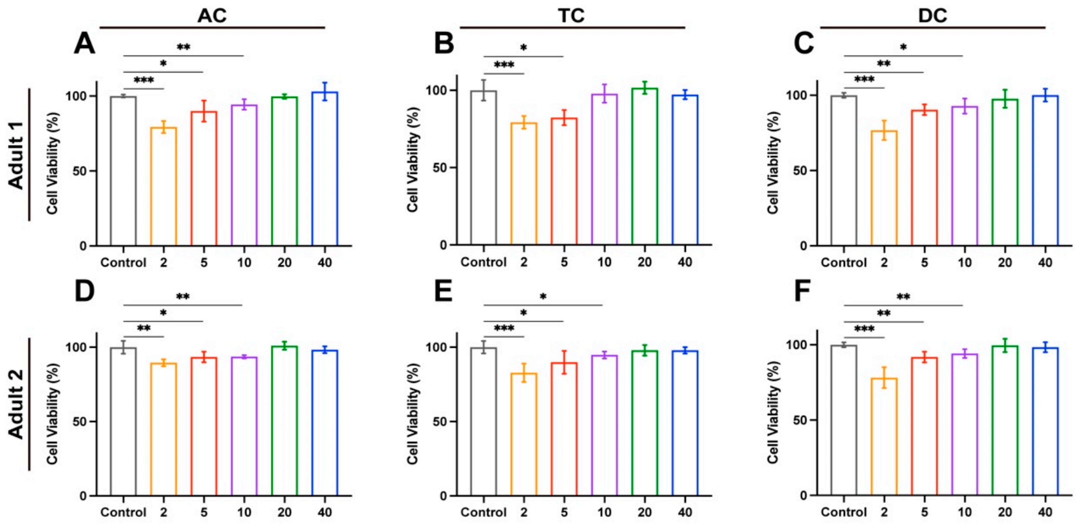

Fig. S6. Effect of SHIME supernatant on cell viability of Caco-2/HT29-MTX-E12 co-culture cell model in the control group. **A-C**: Effect of medium containing different dilutions (dilute in ratios of 2:1, 5:1, 10:1, 20:1 and 40:1) of SHIME supernatant from adult 1 AC (A), TC (B) and DC (C) on cell viability in the co-culture cell model. **D-F**: Effect of medium containing different dilutions (dilute in ratios of 2:1, 5:1, 10:1, 20:1 and 40:1) of SHIME supernatant from adult 2 AC (D), TC (E) and DC (F) on cell viability in the co-culture cell model. Data are show as mean ± SD (n=6). Statistical significance was calculated by Student's t-test (two-tailed). * $p < 0.05$; ** $p < 0.01$; *** $p < 0.001$.

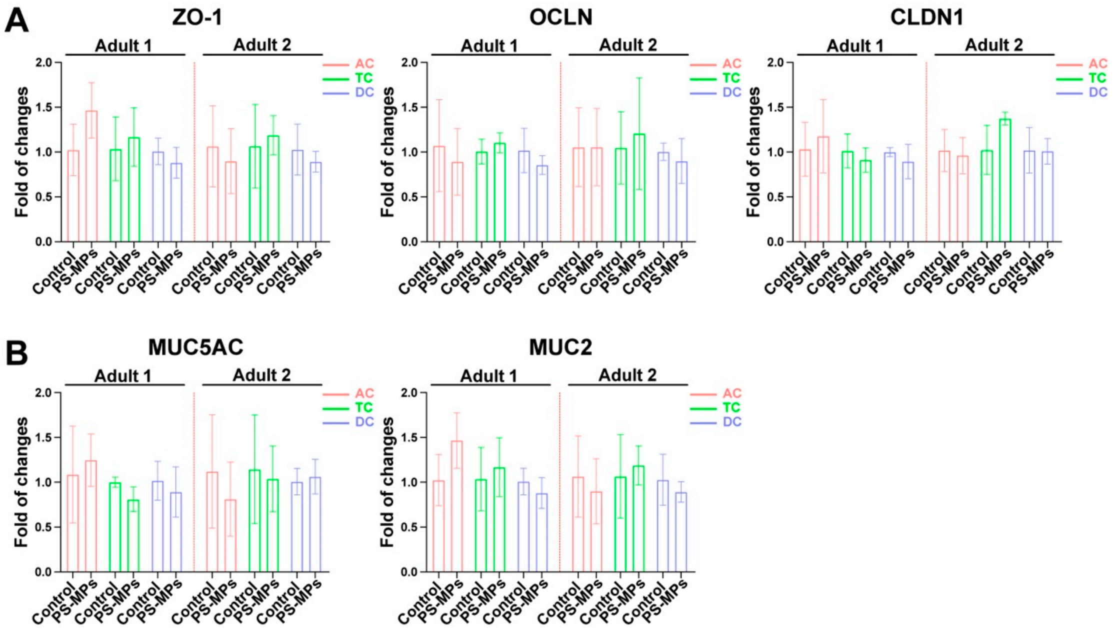

Fig. S7. qRT-PCR quantification of intestinal function genes in Caco-2/HT29-MTX-E12 co-culture cell. **A**: Relative mRNA expression of tight-junction related ZO-1, OCLN and CLDN1 genes. **B**: Relative mRNA expression of mucus secretion related MUC5AC and MUC2 genes. Data are shown as mean ± SD (n=3). Statistical significance was calculated by Student's t-test. * $p < 0.05$; ** $p < 0.01$; *** $p < 0.001$.

Table. S1. The primers used in this study.

Gene	Forward primer (5'→3')	Reverse primer (5'→3')
V3-V4	ACTCCTACGGGAGGCAGCAG	GGACTACHVGGGTWTCTAAT
ZO-1	TCCATTGCCCTCGCAGTATG	CTGAATTACCTTCACCATGTGCTC
OCLN	TTGCCATTGTACTGGGGTTCAT	GACACATTTTTAACCCACTCCTCG
CLDN1	TGGTGGTGGGCATCCTCCTG	AATTCGTACCTGGCATTGACTGG
MUC5AC	GGAAAAACGGCATCGTGGTC	TTCCTGTCGTTGGTGCAAGT
MUC2	CAGCACCGATTGCTGAGTTG	GCTGGTCATCTCAATGGCAG
GAPDH	GTGGAAGGACTCATGACCACAG	GCAGGGATGATGTTCTGGAGAG
